# Validation and clinical evaluation of a SARS-CoV-2 surrogate virus neutralisation test (sVNT)

**DOI:** 10.1080/22221751.2020.1835448

**Published:** 2020-11-01

**Authors:** Benjamin Meyer, Johan Reimerink, Giulia Torriani, Fion Brouwer, Gert-Jan Godeke, Sabine Yerly, Marieke Hoogerwerf, Nicolas Vuilleumier, Laurent Kaiser, Isabella Eckerle, Chantal Reusken

**Affiliations:** a Centre for Vaccinology, Department of Pathology and Immunology, University of Geneva, Geneva, Switzerland; b Centre for Infectious Disease Control, WHO COVID-19 reference laboratory, RIVM, Bilthoven, Netherlands; c Department of Microbiology and Molecular Medicine, University of Geneva, Geneva, Switzerland; d Laboratory of Virology, Geneva University Hospitals, Geneva, Switzerland; e Division of Laboratory Medicine, Department of Diagnostics, Geneva University Hospitals and Geneva University, Geneva, Switzerland; f Division of Laboratory Medicine, Department of Medicine, Faculty of Medicine, Geneva, Switzerland; g Division of Infectious Disease, Geneva University Hospitals, Geneva, Switzerland; h Geneva Centre for Emerging Viral Diseases, Geneva University Hospitals, Geneva, Switzerland

**Keywords:** SARS-CoV-2, neutralising antibodies, surrogate virus neutralisation assay, pseudovirus neutralisation assay, cell-based virus neutralisation assay

## Abstract

To understand SARS-CoV-2 immunity after natural infection or vaccination, functional assays such as virus neutralising assays are needed. So far, assays to detect SARS-CoV-2 neutralising antibodies rely on cell-culture based infection assays either using wild type SARS-CoV-2 or pseudotyped viruses. Such assays are labour-intensive, require appropriate biosafety facilities and are difficult to standardize. Recently, a new surrogate virus neutralisation test (sVNT) was described that uses the principle of an ELISA to measure the neutralisation capacity of anti-SARS-CoV-2 antibodies directed against the receptor binding domain. Here, we performed an independent evaluation of the robustness, specificity and sensitivity on an extensive panel of sera from 269 PCR-confirmed COVID-19 cases and 259 unmatched samples collected before 2020 and compared it to cell-based neutralisation assays. We found a high specificity of 99.2 (95%CI: 96.9–99.9) and overall sensitivity of 80.3 (95%CI: 74.9–84.8) for the sVNT. Clinical sensitivity increased between early (<14 days post symptom onset or post diagnosis, dpos/dpd) and late sera (>14 dpos/dpd) from 75.0 (64.7–83.2) to 83.1 (76.5–88.1). Also, higher severity was associated with an increase in clinical sensitivity. Upon comparison with cell-based neutralisation assays we determined an analytical sensitivity of 74.3 (56.4–86.9) and 98.2 (89.4–99.9) for titres ≥10 to <40 and ≥40 to <160, respectively. Only samples with a titre ≥160 were always positive in the sVNT. In conclusion, the sVNT can be used as an additional assay to determine the immune status of COVID-19 infected of vaccinated individuals but its value needs to be assessed for each specific context.

## Introduction

In 2020, the world is facing an unprecedented global health crisis after the emergence of the novel coronavirus severe acute respiratory syndrome coronavirus 2 (SARS-CoV-2), the causative agent of the disease COVID-19. The pandemic spread of this virus immediately raised a demand for serological assays to support clinical and public health management, e.g. to detect a recent or past infection, to assess the level of (sub) population exposure and, to investigate different types of immune responses and levels of potential immunity against re-infection.

Seven months into the outbreak, a plethora of serological assays is available [1] that allows the routine detection of several classes of antibodies, i.e. IgM, IgG and IgA [2–5]. However, to understand immunity after natural infection or vaccination, a functional analysis of the elicited antibody responses, such as avidity for the most immunogenic viral antigens and virus neutralising activity, is of utmost importance [6].

So far, assays to determine SARS-CoV-2 neutralising capability of antibodies rely on handling of wild type or pseudotyped viruses and use cell-culture based infection as a read-out. This requires a biosafety level (BSL) 3 laboratory for wild type SARS-CoV-2, or a BSL-2 laboratory for pseudotyped viruses such as vesicular stomatitis virus (VSV) or lentivirus-based systems. These in-house assays are difficult to standardize across laboratories, especially in the absence of an international standard, as assay characteristics vary depending on culture conditions, virus strains and cell lines used. Furthermore, these assays are labour-intensive, require highly skilled personnel, have a low throughput and results are only available after several days.

Recently, the first commercial assay has become available [7] that indirectly and semi-quantitatively measures the neutralising functionality of SARS-CoV-2 antibodies while overcoming the above limitations. During natural infection, SARS-CoV-2 binds to its cellular receptor, the angiotensin-converting enzyme 2 (ACE2), via the receptor binding domain (RBD) of the viral spike (S) glycoprotein, which is an essential step to establish an infection of the cell [8,9]. The majority, but not all, of the neutralising antibodies are directed against the RBD leading to an inhibition of this interaction [6]. This commercial assay detects SARS-CoV-2 antibodies that competitively inhibit the interaction between recombinant RBD-HRP fusion protein and recombinant ACE2 that is coated on 96-well plates. The assay is independent of the use of replicating or pseudotyped virus and cell cultures and uses the same format/set-up as Enzyme-Linked Immunosorbent Assays (ELISA), allowing for high-throughput, automation and fast turnaround times.

Here, we present an independent, two-centre evaluation of the robustness, specificity and sensitivity of a commercially available version of this novel functional immune-assay based on an extensive panel of sera from (a) a heterogeneous cohort of PCR-confirmed COVID-19 patients, (b) pre-outbreak syndromic patients with respiratory complaints including confirmed recent infections with the four common human coronaviruses (HCoV) and (c) pre-outbreak population sera. The assay performance was evaluated against the conventional cell culture-based wildtype SARS-CoV-2 (Gold Standard method) and VSV-based pseudo-type neutralisation assays to assess its value for measuring levels of functional antibodies directed against SARS-CoV-2.

## Material and methods

### Sample collection

RIVM: sera from common CoV cases and non-CoV respiratory cases were partially obtained from a previous study at the National Institute of Public Health and the Environment (METC Noord-Holland, http://www.trialregister.nl; NTR3386 and 4818 [10]) and partially from anonymized leftover serum from routine diagnostics for respiratory pathogens or SARS-CoV-2. The current study was performed in accordance with the guidelines for sharing of patient data for observational scientific research in emergency situations as issued by the Commission on Codes of Conduct of the Federation of Dutch Medical Scientific Societies (https://www.federa.org/federa-english).

University of Geneva/HUG: Anonymized leftovers of serum and plasma samples were used for this analysis. Ethical approval for all samples used in this study was waived by the local ethics committee of the HUG that approves usage of leftover of patient samples collected for diagnostic purposes in accordance with our institutional and national regulations.

The study included blood samples from 269 real-time (RT)-PCR confirmed COVID-19 cases (sensitivity panel) and 259 unmatched samples collected before 2020 (specificity panel). We used days post onset of symptoms (dpos) in cases where the onset was known or days post PCR diagnosis (dpd) if the onset was unknown. Samples were stored at −20°C and thawed immediately before the assay was performed. The specificity panel included sera collected before 2020 from: (i) healthy blood donors (*n *= 100); (ii) patients with PCR-confirmed common HCoV infections two months prior, i.e. HCoV-229E (*n *= 12), HCoV-NL63 (*n *= 10), HCoV-HKU1 (*n *= 6) or HCoV-OC43 (*n *= 10) [10]; (iii) patients with a recent PCR-confirmed non-CoV respiratory infection (*n *= 14), i.e. Influenza A virus (*n *= 3), human metapneumovirus (HMPV) (*n *= 4), respiratory syncytial virus (RSV) B (*n *= 1), RSV A + HMPV (*n *= 1), hemophilus influenza (*n *= 1), mycoplasma pneumonia (*n *= 1) and rhinovirus (*n *= 3); (iv) patients with respiratory complaints that tested negative for a suspected *Bordetella pertussis* infection (*n *= 16); (v) patients with an acute cytomegalovirus (*n *= 10) or acute Epstein–Barr virus (*n *= 10) infection; as well as (vi) adult (*n *= 21) and child (*n *= 50) patients who came for routine diagnostic purposes to the hospital (Table 1). The sensitivity panel included sera from 269 PCR-confirmed COVID-19 patients. Of these sera, 92 were taken before and 177 after 14 dpos/dpd. Severity of disease ranged from asymptomatic (*n *= 3) to mild (non-hospitalized, *n *= 92), severe (hospitalized, *n *= 87) and ICU-admitted/deceased (hospitalized, *n *= 54). For 33 patients the severity of disease was unknown (Table 1).

**Table 1.** Demographics.

						Sex		
Cohort		N	Collection year	Age range		Female	Male	Unknown
Pre-pandemic samples		259						
	Adult patients	21	2018	25–71		15	6	0
	Child patients	50	2018	1–11		24	26	0
	CMV	10	2016	Unknown		0	0	10
	EBV	10	2016	Unknown		0	0	10
	Suspected Pertussis	16	2019	<18		0	0	16
	Other Resp. Diseases	52	2011–2015	>60		0	0	52
	Blood Donors	100	2016	18–79		0	0	100
PCR-confirmed COVID-19 patients		269	2020	24–91		66	136	67
	<14 dpos/dpd	92		24–88		22	59	11
	≥14 dpos/dpd	177		24–91		44	77	56
Severity								
	Asymptomatic	3		57–71		0	3	0
	Mild	92		24–91		29	31	32
	Severe	87		42–88		24	49	14
	ICU/Deceased	54		56–87		8	29	17
	unknown	33		37–83		5	24	4

### Surrogate SARS-CoV-2 virus neutralisation test

The surrogate virus neutralisation test (sVNT) (GenScript cPass™ SARS-CoV-2 Neutralization Antibody Detection Kit, Genscript, The Netherlands) was performed according to the manufacturer's instructions. Both laboratories used the same lot number (20E012157). Briefly, serum samples as well as positive and negative assay controls were diluted 1:10 in sample dilution buffer and mixed with an equal volume of HRP-conjugated RBD. Controls were tested in duplicates and samples in singular. After a 30 min incubation at 37°C, 100 µl of this mixture was transferred to a 96-well plate coated with recombinant ACE2. After incubation at 37°C for 15 min, the supernatant was removed and the plate was washed 4x using the provided wash buffer. 100 µl tetramethylbenzidine substrate was added and incubated for 15 min at room temperature before the reaction was stopped by addition of 50 µl stop solution. Plates were read at 450 nm immediately afterwards. Percentage reduction (%reduction) for each sample was calculated by using the following formula:

$$\text{\%}\mathrm{reduction}=\left(1\;-\frac{\mathrm{OD}450\;(\mathrm{sample})}{\mathrm{Average}\;\mathrm{OD}450\;(\mathrm{neg}.\;\mathrm{ctrl}.)}\right)\;\times 100$$

### VSV-based Pseudovirus Neutralisation Test (PNT_50_)

The VSV-based pseudovirus neutralisation test, was done as described previously [2]. Briefly, African green monkey (VeroE6) cells were seeded in 96-well plates at 2×10^4^ cells per well and grown into confluent monolayer overnight. Sera from patients were inactivated at 56°C for 30 min and diluted from 1:5–1:1280 in DMEM supplemented with 2% foetal bovine serum (FBS). VSV-based SARS-CoV-2 pseudotypes (generated according to Berger, Rentsch, and Zimmer [11] and Torriani et al. [12] expressing a 19 amino acid C-terminal truncated spike protein [13]; NCBI Reference sequence: NC_045512.2) were diluted in DMEM supplemented with 2% FBS in order to have MOI=0.01 per well and added on top of serum dilutions (final serum dilutions obtained were from 1:10–1:2560). The virus-serum mix was incubated at 37°C, for 2 h. Vero E6 cells were then infected with 100 µl of virus-serum mixtures. After incubation at 37°C for 1.5 h, cells were washed once with 1X PBS and DMEM supplemented with 10% FBS was added. After 16–20 h of incubation at 37°C and 5% CO_2_, cells were fixed with 4% formaldehyde solution for 15 min at 37°C and nuclei stained with 1 µg/ml DAPI solution. GFP positive infected cells were counted with ImageXpress® Micro Widefield High Content Screening System (Molecular Devices) and data analyzed with MetaXpress 5.1.0.41 software.

### SARS-CoV-2 virus neutralisation test

SARS-CoV-2 virus neutralisation tests were performed exactly as described by Rijkers et al. [14] Duplicates of two-fold serial dilutions (starting at 1:10) of heat-inactivated sera (30 min, 56°C) were incubated with 100 TCID_50_ of SARS-CoV-2 strain HCoV-19/Netherlands/ZuidHolland_10004/2020 (EVAg cat.nr. 014V-03968) at 35°C and 5% CO_2_, for 1 h in 96-wells plates. Vero-E6 cells were added in a concentration of 2×10^4^ cells per well and incubated for three days at 35°C in an incubator with 5% CO_2_. The serum virus neutralisation titre (VNT_50_) was defined as the reciprocal value of the sample dilution that showed a 50% protection of virus growth. Samples with titres ≥ 10 were defined as SARS-CoV-2 seropositive.

### Wantai SARS-CoV-2 total antibody assay

The Wantai SARS-CoV-2 total antibody ELISA (Beijing Wantai Biological Pharmacy Enterprise, Beijing, China; catalogue number WS1096) was performed exactly according to the manufacturer's instructions [3]. This assay is a double-antigen sandwich ELISA using the recombinant RBD of SARS-CoV-2 as antigen. Optical density (OD) is measured at 450 nm and the antibody titre for each sample is calculated as the ratio of the reading of that sample to the reading of a calibrator (included in the kit): OD ratio.

### Statistical analysis

Statistical analysis was performed with GraphPad Prism version 8.4.3 using Mann–Whitney or Kruskal–Wallis test with Dunn's multiple comparison test where appropriate. Linear regression was also performed using GraphPad Prism version 8.4.3. Calculation of sensitivity, specificity and 95% confidence intervals (95%CI) was done using the VassarStats statistical toolbox (http://www.vassarstats.net/). Results with *p* values <0.05 were considered significant.

## Results

**Figure 1.**
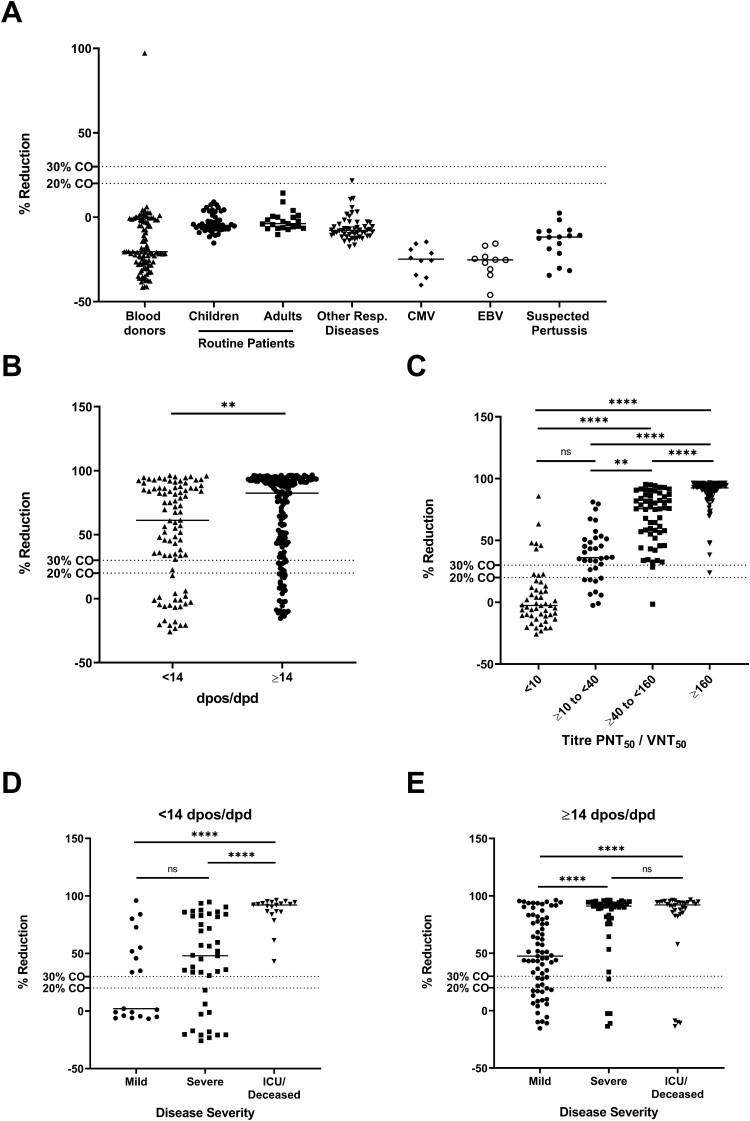
%reduction (inhibition of RBD-ACE2 binding) of samples of the specificity panel. **%**reduction of PCR-confirmed COVID-19 patient samples stratified **(A)** by days post onset of symptoms (dpos) or days post diagnosis (dpd) (**B**), by results of PNT_50_/VNT_50_ titre (**C**) and by disease severity (**D** and **E**). Dashed lines indicates 20% or 30% cut-off (CO).

In this study, we validated an ELISA-based surrogate SARS-CoV-2 neutralisation test (sVNT) using specificity and sensitivity panels as described above. Among the 259 samples in the specificity panel we identified two samples, one among the blood donors and one in the cohort with a recent non-CoV respiratory infection (HMPV infected) that were positive using the manufacturer recommended cut-off of 20% reduction (%reduction: 97.2 and 21.6, respectively). Both samples were tested in the VNT_50_ and did not show any SARS-CoV-2 neutralising activity. Considering an alternative cut-off of 30% reduction, as proposed in a recent publication by the manufacturer [7], only the blood donor sample remained positive for blocking RBD binding to ACE2 (Figure 1(A)). This indicates a specificity of 99.2 (95% CI: 96.9–99.9) and 99.6 (95% CI: 97.5–99.9) for a 20% and 30% cut-off, respectively (Table 2).

**Table 2.** Specificity of sVNT.

		20% Cut-off				30% Cut-off		
Category	Total (n)	Positive (%)	Negative (%)	Specificity (95% CI)		Positive (%)	Negative (%)	Specificity (95% CI)
Pre-pandemic adult patients	21	0 (0)	21 (100)	100 (80.8–100)		0 (0)	21 (100)	100 (80.8–100)
Pre-pandemic child patients	50	0 (0)	50 (100)	100 (91.1–100)		0 (0)	50 (100)	100 (91.1–100)
CMV	10	0 (0)	10 (100)	100 (65.5–100)		0 (0)	10 (100)	100 (65.5–100)
EBV	10	0 (0)	10 (100)	100 (65.5–100)		0 (0)	10 (100)	100 (65.5–100)
Suspected Pertussis	16	0 (0)	16 (100)	100 (75.9–100)		0 (0)	16 (100)	100 (75.9–100)
Other Resp. Diseases	52	1 (1.9)	51 (98.1)	98.1 (88.4–99.9)		0 (0)	52 (100)	100 (91.4–100)
Blood Donors	100	1 (1.0)	99 (99.0)	99.0 (93.8–99.9)		1 (1.0)	99 (99.0)	99.0 (93.8–99.9)
Total	259	2 (0.8)	257 (99.2)	99.2 (96.9–99.9)		1 (0.4)	257 (99.6)	99.6 (97.5–99.9)

CMV, cytomegalovirus; EBV, Epstein-Barr virus; ILI, influenza like illness; 95% CI, 95% confidence interval.

**Figure 2.**
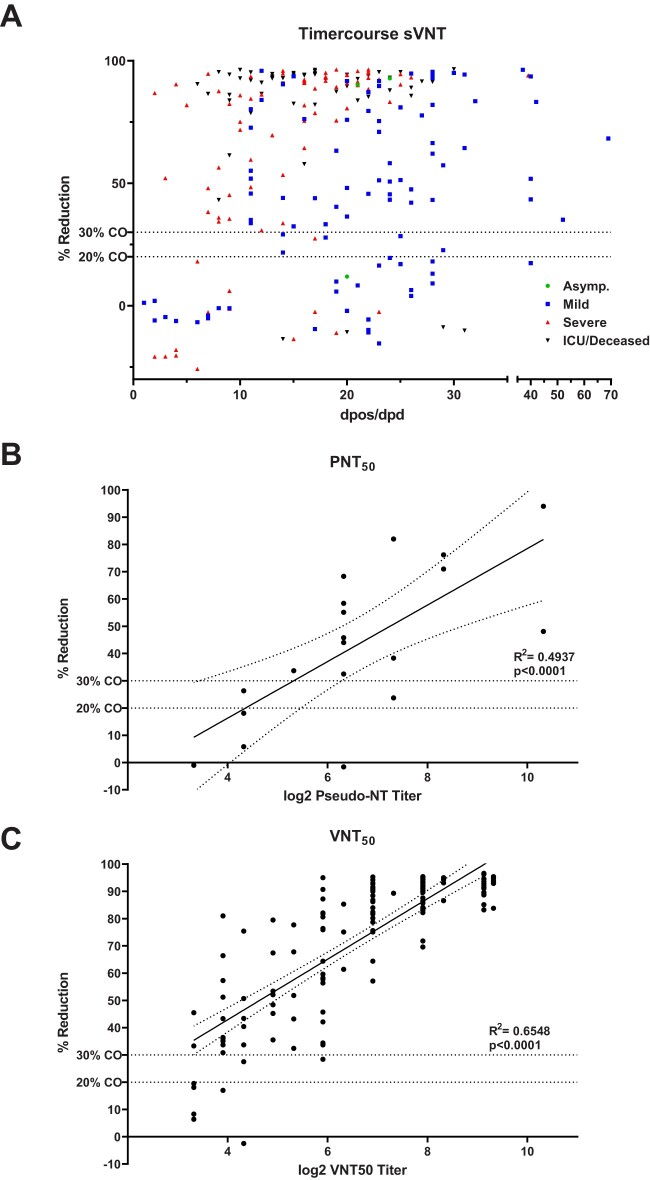
%reduction (inhibition of RBD-ACE2 binding) of COVID-19 patient samples by dpos/dpd and disease severity (**A**). Linear correlation of PNT_50_ (**B**) and VNT_50_ (**C**) endpoint titres with %reduction of RBD-ACE2 binding. Dashed line indicates 20% or 30% cut-off (CO).

Among the 269 sera of confirmed COVID-19 patients in the sensitivity panel, 216 sera tested positive in the sVNT, resulting in an overall clinical sensitivity of 80.3 (95%CI: 74.9–84.8). In sera sampled before 14 dpos/dpd 69/92 samples blocked RBD-ACE2 interaction (clinical sensitivity 75.0 (95%CI: 64.7–83.2) while 147/177 sera sampled at or after 14 dpos/dpd tested positive, resulting in an increased clinical sensitivity of 83.1 (95%CI: 76.5–88.1). In addition, median %reduction significantly increased between <14 and ≥14 dpos/dpd from 61.2% to 82.5% (Mann Whitney test, *p*=0.0019) (Figure 1(B), Figure 2(A) and Table 3). Furthermore, we analysed the relationship between the severity of COVID-19 disease and the clinical sensitivity of the sVNT as stronger immune responses were observed in severe vs mild cases [14,15]. We observed that the assay sensitivity increased with an increased disease severity, being higher in hospitalized patients vs outpatients, regardless of the time of sampling. During the acute phase (<14 dpos/dpd) of the infection, the clinical sensitivity in mild cases was 47.4% and increased to 70.7% and 100% in severe and ICU/deceased patients, respectively (Figure 1(D) and Table 3). A similar observation was made in samples collected ≥14 dpos/dpd, where sensitivity increased from 75.3% in mild cases to 91.3% and 88.2% in severe and ICU/deceased cases, respectively (Figure 1(E) and Table 3). The median %reduction in the sVNT increased with disease severity, but this increase was only significant for mild vs severe and mild vs ICU/deceased cases both before and after 14 dpos/dpd (Kruskal–Wallis test) (Figure 1(D, E)). From three asymptomatic patients, sampled at ≥14 dpos/dpd, two showed a high %reduction of RBD-ACE2 binding while the other one remained negative in the sVNT. When considering the alternative cut-off of 30% reduction to determine positivity in the sVNT, the clinical sensitivity of the assay decreased by 0–9 percentage points but the overall picture remained unchanged (Table 3).

**Table 3.** Sensitivity of sVNT.

			20% Cut-off				30% Cut-off		
Category		Total	Positive (%)	Negative (%)	Sensitivity % (95% CI)		Positive (%)	Negative (%)	Sensitivity % (95% CI)
All		269	216 (80.3)	53 (19.7)	80.3 (74.9-84.8)		207 (77.0)	62 (23.0)	77.0 (71.4-81.8)
dpos/dpd									
	<14 days	92	69 (75.0)	23 (25.0)	75.0 (64.7-83.2)		68 (73.9)	24 (26.1)	73.9 (63.5-82.3)
	≥14 days	177	147 (83.1)	30 (16.9)	83.1 (76.5-88.1)		139 (78.5)	38 (21.5)	78.5 (71.6-84.2)
*P*-NT50/VNT50									
	<10	50	9 (18.0)	41 (82.0)	18.0 (9.0-31.9)		6 (12.0)	44 (88.0)	12.0 (5.0-25.0)
	≥10 to <40	35	26 (74.3)	9 (25.7)	74.3 (56.4-86.9)		24 (68.6)	11 (31.4)	68.6 (50.6-82.6)
	≥40 to <160	57	56 (98.2)	1 (1.8)	98.2 (89.4-99.9)		55 (96.3)	2 (3.7)	96.3 (86.2-99.4)
	≥160	116	116 (100)	0 (0)	100 (96.0-100)		115 (99.1)	1 (0.9)	99.1 (94.6-100)
	ND	11	9 (81.8)	2 (18.2)	81.8 (47.8-96.8)		7 (63.6)	4 (36.4)	63.6 (31.6-87.6)
Severity <14 dpos/dpd									
	Mild	19	9 (47.4)	10 (52.6)	47.4 (25.2-70.5)		9 (47.4)	10 (52.6)	47.4 (25.2-70.5)
	Severe	41	29 (70.7)	12 (29.3)	70.7 (54.3-83.4)		29 (70.7)	12 (29.3)	70.7 (54.3-83.4)
	Deceased	20	20 (100)	0 (0)	100 (80.0-100)		20 (100)	0 (0)	100 (80.0-100)
	unknown	12	11 (91.7)	1 (8.3)	91.7 (59.8-99.6)		10 (83.3)	2 (16.7)	83.3 (50.9-97.1)
Severity ≥14 dpos/dpd									
	Asymptomatic	3	2 (66.7)	1 (33.3)	66.7 (12.5-98.2)		2 (66.7)	1 (33.3)	66.7 (12.5-98.2)
	Mild	73	55 (75.3)	18 (24.7)	75.3 (63.6-84.4)		50 (68.5)	23 (31.5)	68.5 (56.4-78.6)
	Severe	46	42 (91.3)	4 (8.7)	91.3 (78.3-97.2)		41 (89.1)	5 (10.9)	89.1 (75.6-95.9)
	ICU/Deceased	34	30 (88.2)	4 (11.8)	88.2 (71.6-96.2)		30 (88.2)	4 (11.8)	88.2 (71.6-96.2)
	unknown	21	18 (85.7)	3 (14.3)	85.7 (62.6-96.2)		16 (76.2)	5 (23.8)	76.2 (52.5-90.9)

DPOS, days post onset of symptoms; DPD days post diagnosis; *P*-NT50 pseudovirus neutralization test 50% inhibition titer; VNT50 virus neutralization test 50% inhibition titer; ND, not determined; 95% CI, 95% confidence interval.

As the novel assay is meant to be implemented as a surrogate assay for functional cell-based serological methods that measure SARS-CoV-2 neutralising capacity of antibodies, we compared the performance of the sVNT with the conventional VNT_50_ and PNT_50_ assays. The sVNT detected blocking of ACE2 binding activity in nine of 50 sera of confirmed COVID-19 patients that did not show SARS-CoV-2 neutralising activity in the cell-based assays (Table 3). Conversely, only 26/35 sera with a titre in the range of ≥10 to <40 as well as 56/57 sera with a titre in the range of ≥40 to <160 in the cell-based neutralisation assays showed blocking activity in the sVNT. This results in an analytical sensitivity of 74.3 (95% CI: 56.4–86.9) and 98.2 (95% CI: 89.4–99.9), respectively, when compared to conventional in-house neutralisation assays. Only sera (*n *= 116) with a titre of ≥160 in the PNT_50_/VNT_50_ were always positive in the sVNT. The median %reduction in the four titre subgroups significantly increased with rising PNT_50_/VNT_50_ titres from −2.6 (<10) to 36.0 (≥10 to <40), 75.6 (≥40 to <160) and 92.5 (≥160) (Kruskal–Wallis test) (Figure 1(C)). To further investigate the correlation of PNT_50_/VNT_50_ titres with %reduction in the sVNT, a linear regression analysis was performed using all sera for which an endpoint titre was available (*n *= 154). We found a moderate correlation between PNT_50_ and sVNT %reduction (R^2 ^= 0.4937, *p*>0.0001) (Figure 2(B)) and a slightly better correlation for VNT_50_ titres (R^2 ^= 0.6548, *p*>0.0001) (Figure 2(C)).

We also compared overall clinical sensitivity and specificity of the assay between the two centres where the study was conducted (UNIGE and RIVM). Specificity was comparable with only minor differences between the two laboratories. In contrast, clinical sensitivity was markedly lower at UNIGE compared to samples analysed at RIVM (Table 4). However, samples from late time points dpos/dpd where underrepresented in the UNIGE sample set, giving a potential explanation for this discrepancy.

**Table 4.** Comparison of sVNT between sites.

		**UNIGE**				**RIVM**		
		20% Cut-off		30% Cut-off		20% Cut-off		30% Cut-off
Overall Specificity (95% CI)		100 (93.6-100)		100 (93.6-100)		98.9 (95.8-99.8)		99.5 (96.6-99.9)
Overall Sensitivity (95% CI)		70.9 (56.9-81.9)		63.6 (49.5-75.8)		82.7 (76.8-87.4)		80.4 (74.3-85.3)

95% CI, 95% confidence interval.

Last, we investigated the inter-assay variance of the sVNT by testing a representative subset of samples at least five times at different days. Similar coefficients of variation (%CV) were found in both laboratories. We observed a very low %CV in samples with a mean %reduction > 90%, ranging from 0.22–4.63%CV. However, the %CV increased to 3.55–13.73 in samples with a mean %reduction between 50 and 60%, and to 10.39–20.39 in samples with a mean %reduction around 30%. Samples with a mean %reduction <11% gave a high %CV between 67.33 and 216.38 (Table 5).

**Table 5.** Interassay variance of sVNT.

UNIGE						RIVM				
Sample	No. of repeats	Mean % reduction	SD	% CV		Sample	No. of repeats	Mean % reduction	SD	% CV
						001	5	-4.35	3.30	75.82
						002	5	4.10	8.87	216.39
49_neg_2018	5	7.70	5.18	67.33		003	5	10.28	9.09	88.45
30193717	5	30.21	3.14	10.39		004	5	31.66	6.46	20.39
						005	5	32.75	5.00	15.25
30189617	5	57.29	2.03	3.55		006	5	53.76	7.38	13.73
						007	5	54.89	2.47	4.50
						008	5	92.38	4.28	4.63
30175147	5	96.30	0.21	0.22		009	5	93.71	2.63	2.80

SD, standard deviation; CV, coefficient of variation

## Discussion

The importance of serology in clinical and public health management of SARS-CoV-2 is reflected in the huge amount of immune-assays that are currently being developed or have been released on the diagnostic market [1]. However, a vast majority of these tests are simply measuring qualitatively or semi-quantitatively the presence of IgG, IgM and/or IgA and do not address the functionality of the antibody response elicited by a SARS-CoV-2 infection. Functional assays like virus neutralisation tests are essential to address specific questions related to protective immunity after vaccination or natural infection. However, these type of assays are operated based on in-house protocols and lack standardisation across laboratories [16–18]. We performed an independent two-centre clinical evaluation of the GenScript cPass™ test, which is based on the principle of an inhibition ELISA, to assess its value for routine diagnostics and as surrogate functional assay to measure neutralising capability in SARS-CoV-2 elicited antibody responses.

We observed a high specificity of > 99% and an overall clinical sensitivity of 83% in samples taken ≥14 dpos. Within this group of confirmed COVID-19 patients, the clinical sensitivity was 75% in mild patients and 91% in hospitalized patients. Deeks and colleagues performed a Cochrane assessment of 54 studies using commercial immune-assays [5]. They concluded that in confirmed COVID-19 patients sampled in the periods 15–21 dpos and 22–25 dpos, the sensitivities for IgG were respectively 88.2% (95% CI 83.5–91.8) and 80.3% (95%CI 72.4–86.4). While direct comparison of performances of different serology assays is complicated by differences in test set-up (e.g. testing for all isotypes vs for IgG only, the use of different antigens, measuring antigen binding vs prevention of RBD-ACE2 binding) and differences in patient cohorts used, our results seem to indicate that the sVNT might be a more powerful tool for cohort and population studies than for individual diagnosis of past SARS-CoV-2 infection as the observed sensitivities are in the lower end of the range of average test performances observed by Deeks.

In our study, % inhibition measured by the sVNT showed a moderate correlation with PNT_50_ and VNT_50_ titres with R^2^ values of 0.4937 and 0.6548. This is considerably lower than in the study by Tan et al. which reported an R^2^ of 0.8374 for a pseudovirus-based assay and 0.8591 for a conventional virus neutralisation assay [7]. A potential explanation for this discrepancy is that in the study by Tan et al. a titre was determined by calculating the half-maximum inhibitory concentration while we used %reduction as a readout. While titration results in better quantification of the neutralising antibody response and therefore potentially to a higher degree of correlation, our goal was to evaluate the sVNT how it will most likely be used in a diagnostic laboratory. There, in the majority of cases a titration will not be performed for every sample due to cost reasons.

With respect to the sVNT as an alternative for conventional virus culture-based assays measuring neutralising activity, we observed that on the one hand the sVNT misses samples that have a low virus neutralisation titre in these assays and on the other hand identifies samples as positive that were negative in a Gold Standard tests. A limitation of the sVNT is that it does not measure all neutralising antibodies but only the ones directed against the RBD. It has been shown that SARS-CoV-2 infection does not only induce antibodies against the RBD or the S1 domain, but also against the S2 domain as well as against N [19]. While antibodies directed against the N protein are most likely non-neutralising, antibodies directed against the N-terminal domain of S1 (outside of the RBD) have shown neutralising potential [20]. In addition, for SARS-CoV-1, antibodies directed at the S2 domain show neutralising capabilities [21]. Although S2 domain-mediated neutralisation has yet to be confirmed for SARS-CoV-2, assessing the neutralising ability with the sVNT might indeed miss the presence of virus neutralising capabilities directed outside of the RBD. In addition, another study found a RBD binding monoclonal antibody capable of neutralising SARS-CoV-2 was unable to inhibit RBD binding to ACE2 [22]. Together this leaves substantial room for improvement of the assay by its developers, e.g. by using complete trimerized spike instead of RBD. Furthermore, all of the sera that were reactive in the sVNT but not in the VNT_50_/PNT_50_ were reactive in the Wantai total Ig ELISA that targets RBD as well (data not shown). This indicates that the activity measured in the sVNT is likely due to antibodies directed against the RBD but without a virus neutralising capacity that leads to at least 50% reduction of infected cells. Another explanation for such false positivity with respect to neutralising capability might be the presence of anti-ACE2 autoantibodies as described for specific patient groups [23,24].

The observations of false positives and the observed lower sensitivity (74%) of the sVNT in comparison to conventional tests in samples with neutralising antibody titres ≥10 and <40, indicate that the sVNT cannot fully replace the Gold Standard immune-assays. Depending on the context of use, the lower sensitivity in the range of low virus neutralisation titres might however be acceptable, e.g. for decision making on release of hospitalized patients with virus neutralisation titres above a predetermined threshold from isolation units [25,26]. Nevertheless, as long as the correlates of protection for classical virus neutralisation tests are unknown, the use of the sVNT as functional assay to determine the level of immunity is not warranted.

Besides the aforementioned advantages of the possibility for increased standardisation across laboratories, the possibility for automation, the technical simplicity and the reduced biosafety risk, the sVNT is isotype- and species-independent. Species-independent serology tools are important for research into the epidemiology and ecology of SARS-CoV-2, i.e. for the identification of natural reservoirs and spill-over hosts as well as the monitoring and prevention of human risks from sustained virus circulation in farm animals such as minks [27]. In contrast to virus culture-based assays, the sVNT is at most a semi-quantitative assay making it less valuable as functional assay in immunity studies.

One limitation of our validation was a limited number of sera from patients with other respiratory viral infections, as it is challenging to collect such well-defined convalescent sera of recent PCR-confirmed cases. Thus, although we did not see any cross-reactivity, we cannot definitely exclude it if a larger panel of specimens were to be tested.

In conclusion, the sVNT can be used as an additional assay to estimate the neutralising antibody status of COVID-19 infected of vaccinated individuals, and in cohort studies to confirm results of more routine immuno-assays like IgG, IgM and/or IgA ELISAs and CLIAs. The value of the sVNT as functional assay in patient management, biosafety management, vaccine and immunity studies needs to be assessed for each specific context of use.
